# SYBR Green Real-Time PCR for the Detection of All Enterovirus-A71 Genogroups

**DOI:** 10.1371/journal.pone.0089963

**Published:** 2014-03-20

**Authors:** Audrey Dubot-Pérès, Charlene Y. Q. Tan, Reine de Chesse, Bountoy Sibounheuang, Manivanh Vongsouvath, Koukeo Phommasone, Maël Bessaud, Céline Gazin, Laurence Thirion, Rattanaphone Phetsouvanh, Paul N. Newton, Xavier de Lamballerie

**Affiliations:** 1 Unité Mixte de Recherche_D 190 “Emergence des Pathologies Virales” (Aix-Marseille University, IRD French Institute of Research for Development, EHESP French School of Public Health), Marseille, France; 2 Lao-Oxford-Mahosot Hospital–Wellcome Trust Research Unit, Microbiology Laboratory, Mahosot Hospital, Vientiane, Lao PDR; 3 Centre for Tropical Medicine, Nuffield Department of Clinical Medicine, University of Oxford, Churchill Hospital, Oxford, United Kingdom; 4 Laboratory of Clinical Microbiology, AP-HM Timone, University Hospital Institute for Infectious Disease and Tropical Medicine, Marseille, France; Weill Medical College of Cornell University, United States of America

## Abstract

Enterovirus A71 (EV-A71) has recently become an important public health threat, especially in South-East Asia, where it has caused massive outbreaks of Hand, Foot and Mouth disease every year, resulting in significant mortality. Rapid detection of EV-A71 early in outbreaks would facilitate implementation of prevention and control measures to limit spread. Real-time RT-PCR is the technique of choice for the rapid diagnosis of EV-A71 infection and several systems have been developed to detect circulating strains. Although eight genogroups have been described globally, none of these PCR techniques detect all eight. We describe, for the first time, a SYBR Green real-time RT-PCR system validated to detect all 8 EV-A71 genogroups. This tool could permit the early detection and shift in genogroup circulation and the standardization of HFMD virological diagnosis, facilitating networking of laboratories working on EV-A71 in different regions.

## Introduction

Enterovirus A71 (EV-A71) belongs to the ‘Enterovirus A’ species, Enterovirus genus, *Picornaviridae* family, non-enveloped single strain RNA viruses. EV-A71 and Coxsackievirus A16 (CV-A16) are the main causes of Hand, Foot and Mouth disease (HFMD) outbreaks. HFMD is a common childhood viral infection, usually mild and self-limiting. It is characterised by a brief prodromal fever followed by pharyngitis, mouth ulcers and rash on the hands and feet. Over the last decade HFMD has emerged as a growing public health issue in Asia with an increased burden of severe disease caused by EV-A71, including deaths [1], [2], [3], [4]. Children who died typically presented with a brief febrile illness and subtle neurological signs but then dramatically developed acute refractory cardiac dysfunction and fulminant pulmonary oedema within hours of developing tachycardia, poor peripheral perfusion and tachypnoea [1], [5], [6], [7]. Although severe complicated forms, either cardiac or neurological, only occur in a small minority of children with HFMD, the fulminant course of those who died has caused great public alarm in Asia and justified further attention for the diagnosis and medical management of EV-A71 infections.

**Figure 1:**
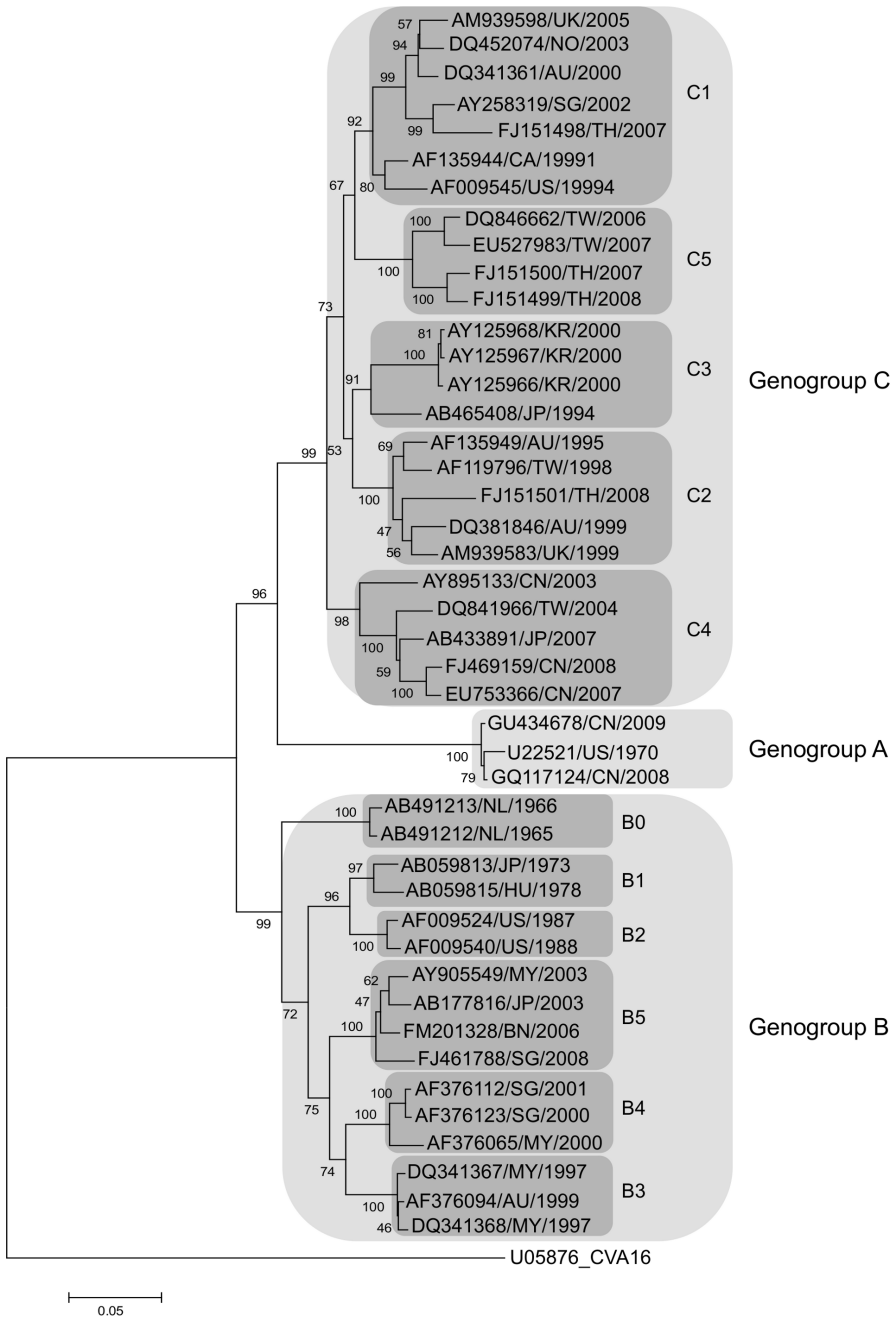
Neighbour-Joining tree of full EV-A71 VP1 sequences. Tree produced using Mega 5.05 software with Kimura-2 model with few full EV-A71 representatives of the 12 subgenogroups: A, B0, B1, B2, B3, B4, B5, C1, C2, C3, C4, C5 aligned using ClustalX 2.1. Bootstrap values (in percentage) were generated by using 1000 replicates. For each strain, the GenBank accession number, the country of origin (ISO 3166 code) and the year are indicated.

The common clinical presentation of uncomplicated EV-A71 infection cannot be distinguished from other enteroviral infections. Biological diagnosis can be achieved using virus isolation onto cell culture followed by neutralisation-based (by using standard sera panels) or VP1 sequencing-based serotyping. Nix *et al.* [8] developed an improved enterovirus typing technique, based on VP1 nested PCR followed by sequencing, which can be used directly from clinical specimens. However, all of these techniques require a few days to be completed and are poorly adapted to the rapid, large-scale EV-A71 diagnosis required for HFMD outbreak investigation. Accordingly, Reverse Transcription-PCRs (RT-PCR) have been developed for the specific detection of EV-A71. In a conventional format [9], [10], [11], [12], [13], [14], [15], the requirement for a post-PCR step (usually an agarose gel electrophoresis) is associated with a high risk of contamination that can seriously jeopardise diagnosis. More recently, the real-time PCR format [16], [17], [18], [19], [20], [21], [22], [23] allows a rapid, specific and sensitive diagnosis with a low risk of contamination. Real-time PCR techniques have been developed in institutions for, and tested almost exclusively on, strains in the populatjons served by these institutions. However, global diversity is wide and EV-A71 strains have been classified into 3 genogroups, (A, B and C) and 12 subgenogroups (A, B0, B1, B2, B3, B4, B5, C1, C2, C3, C4 and C5, Figure 1) on the basis of robust phylogenetic clustering of VP1 nucleotide sequences [24], [25]. Recently five additional genogroups (D, E, F, G, H) have been described [26], [27], [28], [29]. Sequence analysis suggests that most of the existing techniques cannot detect viruses from all subgenogroups. Accordingly, we designed a novel EV-A71 SYBR Green real-time PCR system which is the first to be validated for the molecular detection of all EV-A71 subgenogroups.

## Methods

### Ethics statement

Guardians/parents of HFMD patients (all children) gave informed written consent for their child to be included in HFMD study. This study was granted ethical approval by the Lao National Ethics Committee for Health Research and by the Oxford Tropical Research Ethics Committee. The objective of this study is to determine the incidence, clinical features and responsible enteroviruses amongst paediatric patients with HFMD in Vientiane.

### Primer design

Based on the alignment of all 2,028 EV-A71 VP1 sequences available in GenBank, the amplification primers and probe designed by Tan *et al.* [18] (Table 1) were modified to allow the detection of all EV-A71 sequences.

**Table 1:** EV-A71 primers and probe sequences.

	oligo	5′-3′ sequences	position
Tan *et al.* 2008	Forward primer	GAGAGTTCTATAGGGGACAGT	2466–2489
	Reverse primer	AGCTGTGCTATGTGAATTAGGAA	2669–2647
	probe	6FAM-ACTTACCCAGGCCCTGCCAGCTCC-TAMRA	2498–2521
Modified sequences	Forward primer	GARAGTTCYATAGGRGAYAGY	2466–2489
	Reverse primer	AGCTGTGCTRTGYGARTTRAGRA	2669–2647
	Probe	FAM-ATTGGRGCDTCRTCAAATGCTAGTGA-TAMRA	2595–2620

In the first three lines is the system developed by Tan *et al.* (2008^b^) and below the modified sequences that we used in this article for the detection of EV-A71.

### Synthesis of consensus positive controls for each EV-A71 subgenogroup

All 2,028 full EV-A71 VP1 gene sequences available in GenBank were aligned to produce a consensus sequence for each EV-A71 subgenogroup, delineated by the location of the aforementioned primers. The consensus sequences were secondarily modified between the forward primer and probe sequences to insert the sequence of the *NotI* probe (which includes a *NotI* restriction site, see Ninove *et al.* [30]). This renders the synthetic constructs distinguishable from natural EV-A71 sequences using either TaqMan real-time PCR with the *NotI* probe, amplicon sequencing or *NotI* cleavage. All constructs were obtained using a fusion-PCR procedure based on four overlapping oligonucleotides (59–78 nucleotides in length, Eurogentec (France SASU). The amplicons obtained were then cloned using a standard procedure, the StrataClone PCR cloning kit (Stratagene) and the QIAprep Spin Miniprep Kit (Qiagen) following manufacturers' instructions. The sequence of each plasmid was checked by sequencing and plasmid dsDNA concentrations were measured using a NanoDrop 1000 (Thermo Scientific) and translated into copy numbers. A detailed procedure of each step of the preparation of subgenogroup-specific plasmids is provided in the Supporting Material (Figure S1, Text S1, Table S1).

### Clinical specimens

Throat swabs, vesicle swabs and stool samples collected from patients in the framework of a HFMD study conducted at Mahosot Hospital, Vientiane, Lao PDR (Dubot-Pérès in prep), were used. Children with a new onset of at least one of the following were included: maculopapular or vesicular rash on the palms and/or soles; vesicles or ulcers in the mouth or herpangina.

When possible, EV was isolated onto MRC-5, MA-104, or BGM cells. In a Biosafety level 3 laboratory, 200 µl of patients' sample were inoculated onto confluent cells in a 12 well plate format. After 1 week at 37°C in a 5% CO2 incubator, cells were scraped and 0.2 ml was passaged onto fresh 12 well plates. For samples with CPE, cells were scraped and 1 ml was passaged onto a fresh 25 cm^2^ flask.

RNA was extracted from 200 µl of patient vesicle, throat swab and stool specimens or cell culture supernatant (after 5 minutes centrifugation at 3,000 rpm) using EZ1 Virus Mini Kit v2.0 (Qiagen) with BioRobot EZ1 workstation and eluted in a 90 µl final volume following manufacturer's instructions.

VP1 amplification and sequencing were performed following the protocol described by Nix *et al.* [8]. The sequences were BLASTed on the NCBI website (blastn) for identification.

### PCR optimisation

TaqMan Real-time PCRs were performed using 25 µl of Fast qPCR MasterMix (Eurogentec) in a final volume of 50 µl, including 5 µl of DNA template. The thermal profile used was 2 minutes at 50°C, 5 minutes at 95°C, 45 cycles of 15 seconds at 95°C, 30 seconds at Tm and 30 seconds at 60°C.

SYBR Green real-time amplifications were performed using the one-step Quantitect SYBR Green RT-PCR kit (Qiagen) in a final volume of 25 µl, including 5 µl of template. The thermal profile was 30 minutes at 50°C, 15 minutes at 95°C, 45 cycles of 15 seconds at 94°C, 30 seconds at Tm, 30 seconds at 72°C, ending with a melting curve from 60°C to 90°C. It included the reverse transcription step whatever the nature of the nucleic acid template (DNA for plasmids, RNA for clinical samples).

All real-time amplifications were run on a CFX96 Touch Real-Time PCR system (Bio-Rad).

## Results

### Consensus plasmids for all EV-A71 subgenogroups

Constructs including the VP1 gene region targeted by Tan *et al.* [18] and the sequence of the *NotI* probe were obtained for 12 EV-A71 subgenogroups (A, B0, B1, B2, B3, B4, B5, C1, C2, C3, C4, C5), cloned and quantified (see Tables 2 and 3). At the time we prepared and performed the PCR validation, the VP1 sequences of EV-A71 from genogroups D, E, F, G and H were not available. Serial dilutions of plasmids in molecular grade water were used as templates for real-time PCR optimisation.

**Table 2:** Consensus sequences, including *NotI* site, for 12 EV-A71 subgenogroups.

Subgenogroup	5′→3′ Sequence (NotI probe)
A	GAGAGCTCTATAGGAGATAGTGTGAGTAAGGCCCTCACCCAAGCTTTACCTGCACCCACAGGCCAAAACACCCAAGTGAGCAGTCATCGCTTAGACACTGGAAAAATTATAGCGGCCGCTTATTACGAAATCGGAGCTTCGTCGAATGCTAGTGATGAGAGTATGATTGAGACTCGGTGTGTTCTTAACTCACATAGCACAGCT
B0	GAGAGTTCTATAGGGGACAGTGTGAGTAGAGCACTCACCCAGGCCCTGCCAGCACCCACAGGCCAGAACACACAGGTGAGCAGTCACCGACTGGACACTGGCAAAATTATAGCGGCCGCTTATTATGAAATTGGGGCGTCGTCAAATGCTAGTGACGAGAGCATGATTGAAACGCGATGCGTTCTCAACTCACATAGCACAGCA
B1	GAGAGTTCTATAGGGGACAGTATGAGTAGAGCACTTACTCAGGCCCTGCCAGCACCCACAGGTCAAAACACACAGGTGAGCAGTCATCGACTGGATACTGGCGAAATTATAGCGGCCGCTTATTATGAAATTGGGGCATCGTCAAACACTAGTGACGAGAGTATGATTGAAACACGATGCGTTCTTAACTCACATAGCACAGCA
B2	GAGAGCTCTATAGGAGATAGTGTGAGTAGAGCACTTACCCAGGCCCTGCCAGCACCCACAGGTCAAAACACACAGGTGAGCAGTCATCGACTGGATACTGGCGAAATTATAGCGGCCGCTTATTATGAAATTGGGGCATCGTCAAATACTAGTGACGAGAGTATGATTGAAACACGATGCGTTCTTAACTCACACAGCACAGCA
B3	GAGAGCTCTATAGGAGATAGTGTGAGTAGAGCACTTACCCAGGCCCTGCCAGCACCCACAGGTCAAAACACACAGGTGAGCAGTCATCGACTAGACACTGGCGAAATTATAGCGGCCGCTTATTATGAAATTGGGGCATCGTCAAATACTAGTGATGAGAGTATGATTGAAACACGGTGCGTTCTTAACTCACACAGCACAGCA
B4	GAGAGCTCTATAGGAGATAGTGTGAGTAGGGCACTTACCCAGGCCCTGCCAGCTCCAACAGGTCAGAACACGCAGGTGAGCAGTCATCGACTAGACACTGGTGAAATTATAGCGGCCGCTTATTATGAAATTGGGGCATCGTCAAATACTAGTGATGAGAGTATGATTGAGACACGATGCGTTCTTAATTCACACAGTACGGCA
B5	GAGAGCTCTATAGGAGACAGTGTGAGTAGGGCACTCACCCAGGCCCTGCCAGCACCCACAGGTCAAAACACACAGGTGAGCAGCCATCGATTAGACACCGGTGAAATTATAGCGGCCGCTTATTATGAGATCGGGGCATCATCAAATACTAGTGATGAGAGTATGATTGAGACACGATGCGTCCTTAACTCACACAGTACAGCA
C1	GAGAGTTCTATAGGGGATAGTGTGAGCAGAGCTCTCACCCAAGCTTTACCAGCACCCACAGGCCAAAACACGCAAGTAAGCAGCCACCGGTTGGACACTGGTAAAATTATAGCGGCCGCTTATTATGAAATTGGAGCATCATCAAATGCTAGTGACGAGAGTATGATTGAGACACGGTGTGTTCTTAATTCGCACAGCACAGCT
C2	GAGAGTTCTATAGGGGACAGTGTGAGCAGAGCCCTCACCCGAGCTCTACCGGCACCTACAGGCCAAAACACGCAGGTAAGCAGCCATCGATTGGATACTGGTAAAATTATAGCGGCCGCTTATTATGAAATTGGAGCATCATCAAATGCTAGTGATGAGAGTATGATTGAGACGCGATGTGTTCTTAATTCACATAGCACAGCT
C3	GAGAGTTCTATAGGGGATAGTGTGAGCAGAGCCCTTACCCAAGCTCTACCGGCACCCACAGGCCAGAACACACAGGTGAGCAGTCATCGATTAGATACTGGTAAGATTATAGCGGCCGCTTATTATGAAATTGGAGCATCATCGAATGCTAGTGATGAGAGCATGATTGAGACACGATGTGTTCTTAATTCACACAGTACAGCT
C4	GAAAGTTCCATAGGAGATAGTGTGAGCAGAGCCCTCACTCAAGCTCTACCAGCACCCACAGGTCAGAACACACAGGTGAGCAGTCATCGACTGGATACAGGCAAGATTATAGCGGCCGCTTATTATGAAATTGGAGCATCATCAAATGCTAGTGATGAGAGCATGATTGAGACACGCTGTGTTCTTAACTCGCACAGCACAGCT
C5	GAAAGTTCTATAGGGGACAGCGTGAGCAGAGCCCTCACCCAAGCCCTACCGGCACCTACAGGTCAGAACACGCAGGTAAGCAGCCACCGACTAGACACTGGTAAAATTATAGCGGCCGCTTATTATGAGATTGGAGCATCGTCAAATGCTAGTGATGAGAGTATGATTGAGACACGGTGTGTTCTTAATTCGCACAGCACGGCT

**Table 3:** Concentration of each subgenogroup plasmid extract.

Plasmid name	Concentration (number of copies/µl)
A	7.9×10^9^
B0	1.7×10^10^
B1	8.2×10^9^
B2	1.3×10^10^
B3	1.2×10^10^
B4	8.8×10^9^
B5	1.8×10^10^
C1	2.3×10^10^
C2	1.1×10^10^
C3	1.2×10^10^
C4	1.4×10^10^
C5	1.8×10^10^

### Primer design

The primers and probe described by Tan *et al.* [18] were modified (Table 1) to be able to detect all the EV-A71 sequences, based on *in silico* analysis. Five degenerations were added to the forward primer, R, Y, R, Y and Y at positions 3, 9, 15, 18 and 21, respectively. Five degenerations were added to the reverse primer, R, Y, R, R and R at positions 10, 13, 16, 19 and 22, respectively.

### Probe assay

A new probe was tentatively designed (Table 1), 97 nucleotides upstream from the one described by Tan *et al.* [18]. However, TaqMan real-time PCRs using this probe did not detect subgenogroup A (results in Table 4) and the use of probe was dropped for the subsequent tests.

**Table 4:** Results in Ct of the Taqman real-time PCR performed on plasmids A, B0 C1.

Probe	50 nM	100 nM	250 nM
Subgenogroup A	no	no	no
Subgenogroup B0	30.91	31.21	28.42
Gentype C1	32.19	31.75	31.84

Plasmids A, B0 and C1, at 10^−5^ dilution each, were tested with 2 µM of each primer at Tm 50°C and different concentrations of probe.

### Primer concentration optimisation

Subsequent tests were performed using a SYBR Green real-time assay, 10^−5^ dilutions of each plasmid (corresponding to 39.5×10^4^ to 11.5×10^5^ copies per tube, Table 3) and 0.8 µM, 2 µM or 4 µM of each primers (at Tm 45°C). All combinations of primer concentrations were tested. The Ct values obtained for all conditions are presented in Table 5. The dissociation curve showed only one peak corresponding to the product of amplification. Therefore, Ct value corresponded to the specific amplification.

**Table 5:** Results in Ct of the SYBR Green real-time PCR with various primer concentrations.

Forward primer (µM)	**0.8**	2	4	0.8	**2**	4	0.8	2	**4**
Reverse primer (µM)	**0.8**	0.8	0.8	2	**2**	2	4	4	**4**
Plasmids (C)									
A (4×10^5^)	**32.37**	29.27	27.26	29.55	**26.44**	25.51	29.45	25.93	**24.23**
B0 (8.5×10^5^)	**33.19**	27.56	26.53	29.70	**26.03**	24.04	26.52	24.24	**22.58**
B1 (4.1×10^5^)	**30.32**	28.39	28.09	28.56	**26.76**	26.37	27.60	26.79	**26.14**
B2 (6.5×10^5^)	**29.89**	27.28	26.23	28.15	**25.10**	23.47	26.48	23.93	**23.12**
B3 (6×10^5^)	**32.10**	28.20	26.20	27.86	**25.60**	24.61	28.02	25.38	**24.33**
B4 (4.4×10^5^)	**33.88**	30.54	29.32	30.22	**27.66**	26.62	30.02	27.03	**25.73**
B5 (9×10^5^)	**30.91**	25.51	23.54	26.52	**22.68**	21.15	25.40	22.14	**20.59**
C1 (1.2×10^6^)	**33.09**	28.50	25.69	30.01	**26.03**	24.09	28.10	25.13	**23.51**
C2 (5.5×10^5^)	**33.02**	29.78	27.11	28.41	**26.07**	25.42	27.35	25.30	**24.01**
C3 (6×10^5^)	**30.25**	27.33	26.31	25.60	**23.40**	22.73	24.25	22.20	**21.97**
C4 (7×10^5^)	**31.16**	26.82	25.48	26.48	**22.79**	21.87	24.75	21.74	**20.78**
C5 (9×10^5^)	**31.57**	27.76	26.56	27.21	**23.82**	23.00	25.37	22.35	**21.71**

All plasmids, at 10^−5^ dilution each, were tested at Tm 45°C. ‘C’: number of plasmid copies use as template.

For all subgenogroups, the combination of two different concentrations of forward and reverse primer did not provide better amplification than the two primers at the highest concentration. The rise of the primers concentration from 0.8 µM to 4 µM improved the rate of PCR amplification for all subgenogroups. The best improvement was observed for genotype B0 (difference in Ct of 10.61). The lowest difference was observed for genotype B1 (difference in Ct of 4.18).

In average, for all subgenogroups, the Ct decrease was 6.6 when primer concentration was increased from 0.8 µM to 2 µM. The additional Ct decrease provided by primers at 4 µM was 2. Accordingly, the 2 µM primer concentration was chosen for subsequent tests.

### Tm optimisation

SYBR Green real-time PCRs with 2 µM of primers were performed on dilution 10^−5^ of all plasmids with a Tm gradient, 44.6°C, 50.2°C and 55°C (results in Table 6).

**Table 6:** Results in Ct of SYBR Green real-time PCR with Tm 44.6°C, 50.2°C and 55°C.

Plasmids (C)	Tm 44.6°C	Tm 50.2°C	Tm 55°C
A (4×10^5^)	25.21	**24.21**	24.30
B0 (8.5×10^5^)	26.21	23.86	**23.80**
B1 (4.1×10^5^)	24.63	**22.93**	23.80
B2 (6.5×10^5^)	23.25	**22.45**	25.93
B3 (6×10^5^)	23.27	**22.49**	24.89
B4 (4.4×10^5^)	**24.87**	24.88	29.33
B5 (9×10^5^)	25.31	**23.90**	26.37
C1 (4×10^6^)	26.36	**24.36**	25.93
C2 (5.5×10^5^)	24.65	**23.05**	23.64
C3 (6×10^5^)	25.01	**22.89**	25.14
C4 (7×10^5^)	23.53	**22.76**	25.16
C5 (9×10^5^)	26.28	**23.40**	24.33

All plasmids, at 10^−5^ dilution each, were tested with 2 µM of each primer. Cells filled in bold indicate lowest Ct value for each plasmid. ‘C’: number of plasmid copies used as template.

For all subgenogroups, Tm 50.2°C provided Ct values lower (most of subgenogroups) or equivalent (subgenogroups B0 and B4) to those provided by other temperatures. Tm 50°C was chosen for subsequent tests.

### Limit of detection

**Figure 2:**
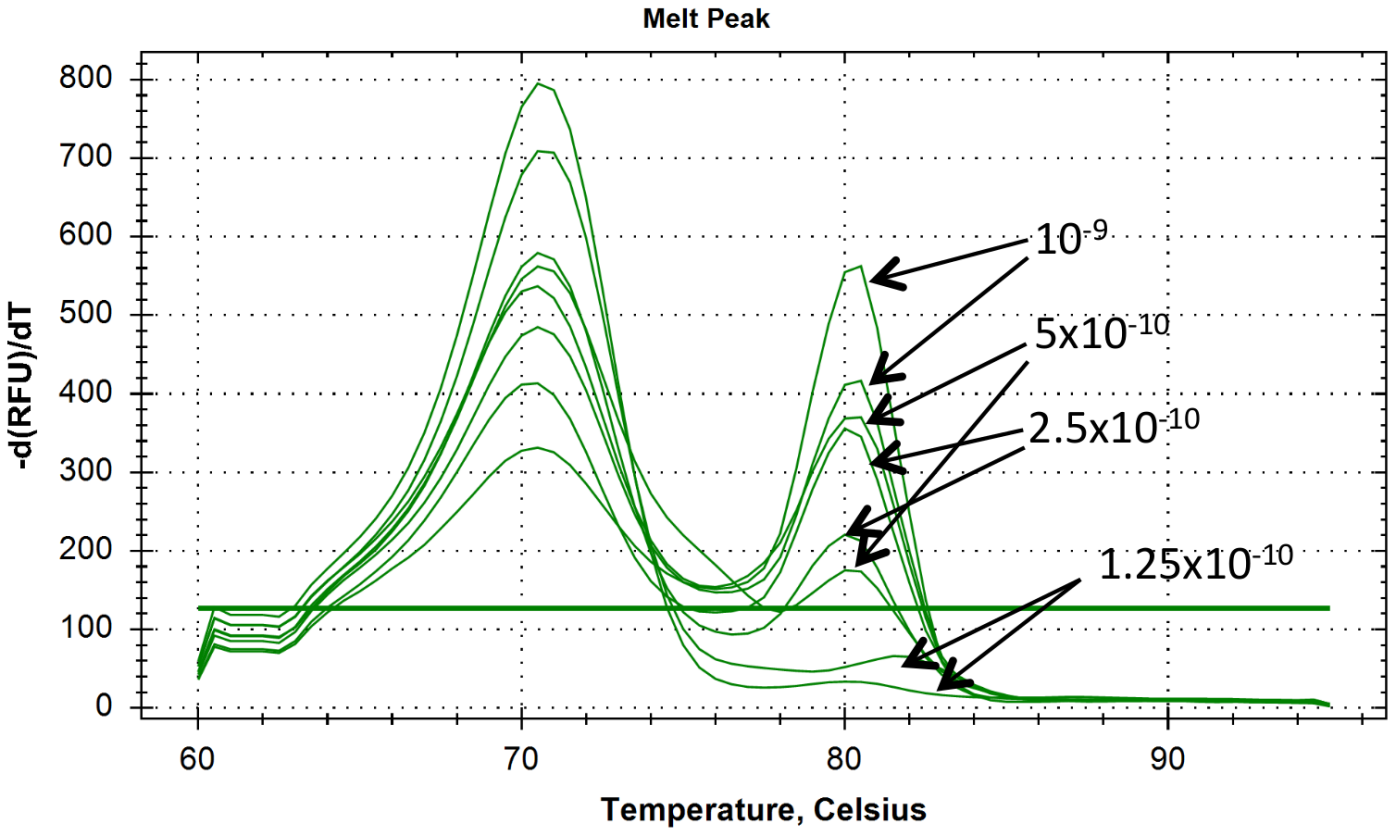
Dissociation curves of the EV-A71 SYBR Green RT-PCR performed on dilutions of plasmid B1.

SYBR Green real-time PCR with 2 µM of primers at Tm 50°C was performed in duplicate using dilutions 10^−9^, 5×10^−10^, 2.5×10^−10^, 1.25×10^−10^, 6.25×10^−11^, 3.13×10^−11^, 1.56×10^−11^ for all subgenogroups (results in Table 7). The dissociation curves obtained with the RT-PCR on plasmid B1 dilutions is shown in Figure 2. Dissociation curves obtained for the other plasmids were similar (not shown).

**Table 7:** Results of SYBR Green real-time PCR on serial dilutions of each plasmids.

dilutions	2.5×10^−9^		10^−9^		5×10^−10^		2.5×10^−10^		1.25×10^−10^		6.25×10^−11^	
Subgeno groups	C	R	C	R	C	R	C	R	C	R	C	R
A	98	+	**39**	**+**	19	−	9	−	4	−		
		+		**+**		−		−		−		
B0	212	+	85	+	**42**	**+**	21	+	10	−	5	−
		+		+		**+**		−		−		−
B1	102	+	41	+	20	+	**10**	**+**	5	−		
		+		+		+		**+**		−		
B2	162	+	**65**	**+**	32	+	16	−	8	+	4	−
		+		**+**		−		−		−		−
B3	150	+	60	+	**30**	**+**	15	+	7	−	3	−
		+		+		**+**		−		−		−
B4	110	+	**44**	**+**	22	−	11	−	5	−		
		+		**+**		−		−		−		
B5	225	+	90	+	45	+	22	+	11	+	**5**	**+**
		+		+		+		+		+		**+**
C1	287	+	115	+	**57**	**+**	28	+	14	−	7	−
		+		+		**+**		−		−		−
C2	137	+	55	+	27	+	**13**	**+**	6	−	3	−
		+		+		+		**+**		−		−
C3	150	+	60	+	**30**	**+**	15	−	7	−	3	−
		+		+		**+**		−		−		−
C4	175	+	**70**	**+**	35	+	17	−	8	−	4	−
		+		**+**		−		−		−		−
C5	225	+	**90**	**+**	45	+	22	+	11	−	5	−
		+		**+**		−		−		−		−

Real-time PCR were performed with 2 µM of primers at Tm 50°C. Cells filled in bold indicate the biggest dilution for which both duplicate are PCR positive ( = limit of detection). ‘C’: number of plasmid copies use as template. ‘R’: PCR result, ‘+’: positive, ‘−’: negative.

The limit of detection was defined as the last dilution positive for both duplicates. The limit of detection was between 5 copies, for subgenogroup B5, and 90 copies, for genogroup C5 with an average of 41 copies.

### Tests on patient samples

**Figure 3:**
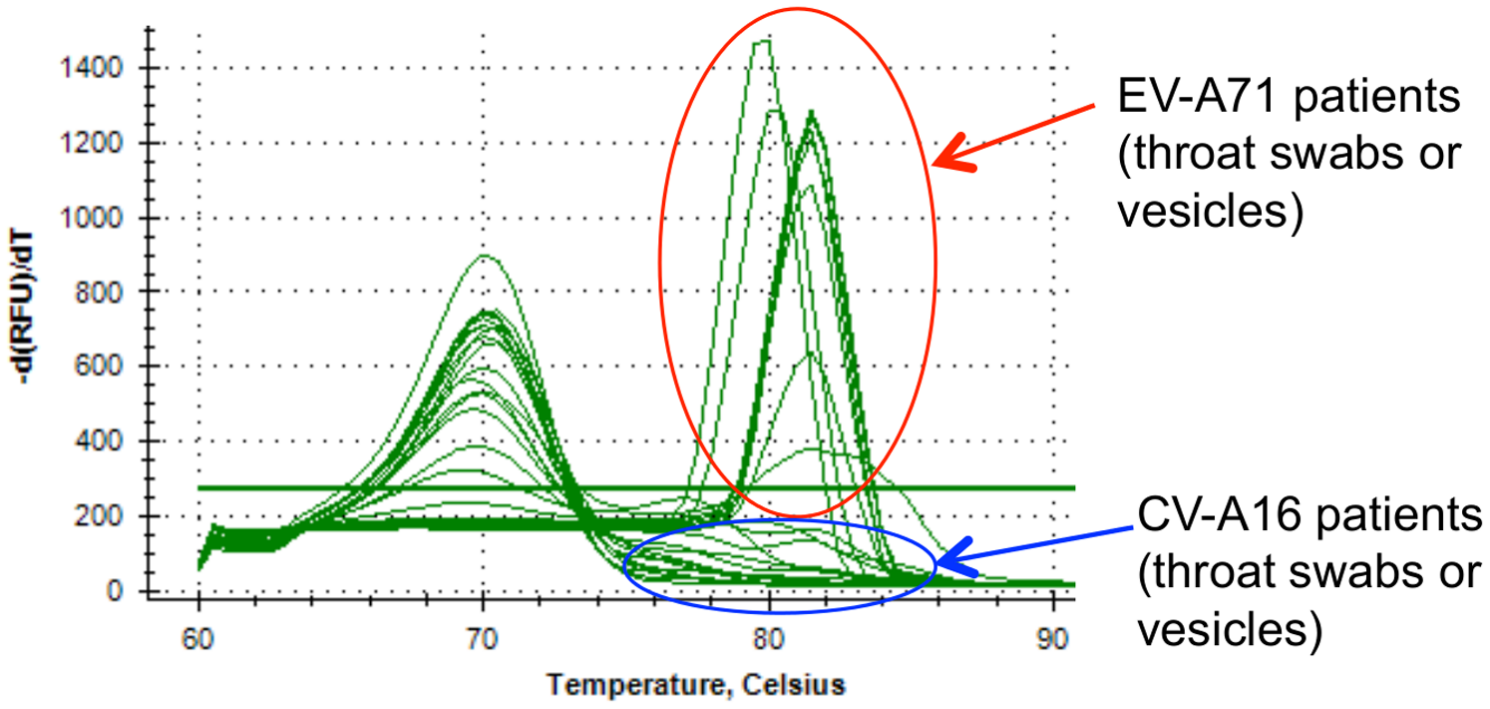
Dissociation curves of EV-A71 SYBR Green RT-PCR performed on HFMD patient specimens (throat swabs or vesicles). All peaks above the threshold line were from EV-A71 patients (confirmed by sequencing).

Specimen from HFMD patients from Vientiane, Lao PDR, were used to test our one-step SYBR Green real-time RT-PCR detection system. The enteroviruses infecting these patients were characterised by sequencing of the VP1 gene. EV-A71 SYBR Green real-time RT-PCR with 2 µM of primers and Tm 50°C was performed on extracts from 23 specimens of 14 patients infected by EV-A71 and from 21 specimens of 17 patients infected by other enteroviruses (2 CV-A6, 15 CV-A16). All specimen from EV-A71 patients were positive by SYBR Green real-time PCR (result confirmed by the sequencing of the PCR products), and all specimens from patients infected by CV-A6 or CV-A16 were negative (Table 8). Dissociation curves obtained for 22 patients (9 EV-A71 positive patients and 13 CV-A16 patients) are shown in Figure 3 (dissociation curves obtained for the other patients were similar, not shown).

**Table 8:** Results of SYBR Green real-time PCR performed on patient and culture samples.

Number of patients	Sample type	EV Serotype	EV species	EV-A71 PCR result	
				Positive specimens	Negative specimens
14	10 throat swabs, 8 vesicles, 5 stools	EV-71	A	23	0
15	8 throat swabs, 10 vesicles, 1 stool	CV-A16	A	0	19
2	2 throat swabs	CV-A6	A	0	2
9	Cell culture	CV-A16	A	0	9
2	Cell culture	CV-B4	B	0	2
4	Cell culture	CV-A24	C	0	4
1	Cell culture	E-18	B	0	1
1	Cell culture	CV-AC13	C	0	1
1	Cell culture	CV-A9	B	0	1
1	Cell culture	CV-B2	B	0	1
1	Cell culture	E-11	B	0	1
1	Cell culture	E-13	B	0	1
1	Cell culture	E-14	B	0	1
1	Cell culture	E-18	B	0	1
1	Cell culture	E-3	B	0	1
1	Cell culture	E-30	B	0	1
1	Cell culture	E-7	B	0	1
1	Cell culture	E-9	B	0	1
1	Cell culture	EV-B75	B	0	1

Extracts from EV isolated on cell culture were also tested by our EV-A71 SYBR Green assay. The viruses tested were CV-A16 from Enterovirus A species, CV-A9, CV-B2, E-11, E-13, E-14, E-18, E-3, E-30, E-7, E-9, EV-B75, and CV-B4 from Enterovirus B species, and CV-A24 and CV-A13 from Enterovirus C species. None of these cultures provided a positive result when tested by the EV-A71 SYBR Green assay (Table 8).

## Discussion

EV-A71 was first recognised as causing large outbreaks of HFMD associated with neurological disease and alarming fatalities in Sarawak, East Malaysia, in 1997 and in Taiwan in 1998 [1], [2]. In 1998, Taiwan experienced the largest outbreak of EV-A71 recorded, with 129,106 cases reported and 405 children with severe complications, 78 of whom died. Further EV-A71 outbreaks occurred in Taiwan in 2000 and 2001. There were 291 severe enteroviral cases, 41 of whom died in 2000, and 393 severe enteroviral cases including 58 deaths in 2001 [31], [32]. In 2008 the CDC in Taiwan reported a total of 317 patients with confirmed severe enteroviral disease including 10 deaths whilst in China a total of 488,955 HFMD cases, 126 of which were fatal, were reported nationwide in 2008 [33], [34]. In addition to these very large outbreaks, many areas, including Japan, Sarawak, Singapore, Taiwan, and Vietnam, have experienced cyclical epidemics occurring every 2–3 years [32], [35], [36], [37], [38]. Outside the Asia-Pacific region EV-A71 is circulating at low levels in Africa, USA and Europe. Between April and July 2012, an outbreak of severe form of HFMD (including deaths) caused by EV-71 was reported in Cambodia (Global Alert and Response, WHO, 13 July 2012)

Early aetiological diagnosis of HFMD is important and allows prevention and control measures to reduce epidemic spread. EV-A71 laboratory diagnosis is also of great diagnostic importance for patients who do not necessarily present with typical HFMD signs.

No difference in virulence has been reported between the EV-A71 subgenogroups but spatiotemporal divergences and shifts in subgenogroup dominance have been reported [39]. In Vietnam, since 2005 the major EV-A71 subgenogroup circulating has been C5 with co-circulation of C1 and C4 [40]. In 2006, subgenogroup C5 emerged in Taiwan. In 2008 and 2009 major outbreaks of subgenogroup C4 were reported in China [14], [34] and C4 is currently circulating in China, Japan, Taiwan, Thailand, Republic of Korea and Vietnam. In 2000, the Republic of Korea recorded a subgenogroup C3 cluster when B4 was dominant elsewhere [41]. Shifts in subgenogroup dominance have been reported in Sarawak and Vietnam [35], [40], [42]. In 2003, Deshpande *et al.* [43] described a fourth genogroup (D), with partial VP1 sequence from a EV-A71 detected in India from an patient with flaccid paralysis. Recently, two EV-A71 strains from Africa (Central African Republic and Cameroun) have been reported belonging to a fifth genogroup (E) [27], [28]. Bessaud *et al.* [26] have shown that other strains circulating in India belong to 3 other distinct genogroups (F, G and H).

Since several subgenogroups are circulating at the same time in the same area and subgenogroup shifts are observed, a diagnosis test able to detect all subgenogroup is essential to avoid failing to detect a EV-A71 strain newly introduced in an area.

Several real-time PCR systems for the detection of EV-A71 have been developed [16], [17], [18], [19], [20], [21], [22], [23]. Nevertheless, only two systems [20], [23] involve degenerated primers to take into account the genetic variability of EV-A71 strains, and all of them were only tested on local EV-A71 strains from HFMD outbreaks.

For the first time we present here a one-step real-time PCR system that has been designed *in silico* to be able to amplify all EV-A71 subgenogroups (primers modified from Tan *et al.* [18]), and tested on 12 subgenogroup consensus sequences (A, B0, B1, B2, B3, B4, B5, C1, C2, C3, C4 C5). Consensus sequences for genogroups D, E, F, G and H have not been produced. However, at the location of forward and reverse primers, the sequences of strains described in these 5 genogroups are similar to some of those among the 12 produced consensus sequences.

We could not accommodate the design of a single probe with the efficient amplification of all genogroups. However, our primers used in a one-step SYBR Green real-time RT-PCR format permitted detection of all subgenogroups with relevant limits of detection (5 to 90 DNA copies). Due to the high level of degeneration of the primers (each contains 5 degenerated positions) this required increase in the concentration of primers up to 2 µM. When tested on a range of clinical specimens it allowed detection of EV-A71 in diverse specimens (throat swab, vesicle, stool) for all 14 EV-A71 infected patients who were tested. Despite the fact that a systematic challenge against other enterovirus types was not performed, both *in silico* analysis and negative results obtained in preliminary tests using CV-A6, CV-A16, CV-A9, CV-B2, E-11, E-13, E-14, E-18, E-3, E-30, E-7, E-9, EV-B75, CV-B4, CV-A24 and CV-A13 templates, from enterovirus species A, B and C respectively, suggest that the assay has high specificity. In particular, CV-A6 and CV-A16, which are known to be responsible for HFMD outbreaks in Asia, are not detected using our amplification technique.

Interestingly, phylogenetic analyses (data not shown) show that the EV-A71 RT-PCR product sequences grouped with full VP1 sequences of the same genogroup. Therefore, the sequencing of the RT-PCR product could permit to assign the detected EV-A71 to a genogroup. The assignment to a subgenogroup is less obvious.

In conclusion, this one-step SYBR Green real-time RT-PCR protocol is expected to be a simple tool allowing the detection of all EV-A71 subgenogroups and permitting detection of shift in genogroup circulation. It may also be helpful for standardising HFMD diagnosis and facilitating the networking of laboratories working on EV-A71 in different regions. Prospective evaluation in HFMD endemic countries is needed.

## Supporting Information

Text S1 **Preparation of subgenogroup-specific plasmids.**
(DOCX)

Figure S1 **Strategy for the cloning of subgenogroup consensus sequences.** Assembly of 4 overlapping oligonucleotides using Taq polymerase then PCR amplification.
(TIF)

Table S1 **Sequences of the oligonucleotides used for the construction of the twelve subgenogroup specific plasmids.**
(DOCX)
