# Supplementary material for: SYBR Green Real-Time PCR for the Detection of All Enterovirus-A71 Genogroups
Source: PLoS One. 2014 Mar 20;9(3):e89963. doi: 10.1371/journal.pone.0089963 (PMC3961242; doi:10.1371/journal.pone.0089963)
Supplement: Text S1 — Preparation of subgenogroup-specific plasmids. (DOCX) [file pone.0089963.s001.docx]

**Text S1. Preparation of subgenogroup-specific plasmids.**

For each EV-A71 subgenogroup (Table 2), four sequentially overlapping oligonucleotides (59-78 nt) were designed to cover the full consensus sequence (204 nt), Eurogentec (France SASU) and to introduce a *NotI* restriction site. The first and third oligonucleotides were designed in forward direction; and the second and fourth oligonucleotides in reverse direction (Figure S1). In addition, 2 forward and reverse primers flanking the full consensus sequences were used. The sequences of the 4 oligonucleotides and the flanking primers for each subgenogroup are given in the Table S1. The oligonucleotides and primers were used at 10µM.

First, intermediate consensus sequences were obtained by separately ligating the first and second oligonucleotides, and the third and fourth oligonucleotides. Briefly, 0.2 µmol of each oligonucleotide were added to 2.5 U of LongAmp hi-fidelity Taq DNA Polymerase (NEB), 0.2 mmol of dNTP and the buffer provided in a final volume of 50µl. Next, the full consensus sequences was obtained by adding 5µl of each intermediate assembly product to 2.5 U of LongAmp Hi-fidelity Taq DNA Polymerase (NEB), 0.2 mmol of dNTP and the buffer provided with the polymerase in a final volume of 50µl. The thermal profile applied for both reactions was: 5 min at 94ºC, 30 sec at 55ºC, and 30 sec at 72ºC.

Last, the full double-stranded DNA consensus sequence was amplified using the subgenogroup-specific flanking primers. 5 µl of DNA were mixed with 2.5 U of LongAmp Hi-fidelity Taq DNA Polymerase (NEB), 0.2µmol of forward and reverse primers, 0.2 mmol of dNTP and the buffer provided in a final volume of 50µl. The thermal profile applied was: 5 min at 94ºC, 10 cycles (30 sec at 94ºC, 30 sec at 55ºC, 30 sec at 72ºC). The T_m_ and number of amplification cycles were optimised to produce sufficient yield of error-free amplicons. The PCR product was then cloned using the StrataClone^TM^ PCR cloning kit (Stratagene) and the QIAprep Spin Miniprep Kit (Qiagen) following manufacturer’s instructions.
