# Supplementary figures and images for: SYBR Green Real-Time PCR for the Detection of All Enterovirus-A71 Genogroups
Source: PLoS One. 2014 Mar 20;9(3):e89963. doi: 10.1371/journal.pone.0089963 (PMC3961242; doi:10.1371/journal.pone.0089963)

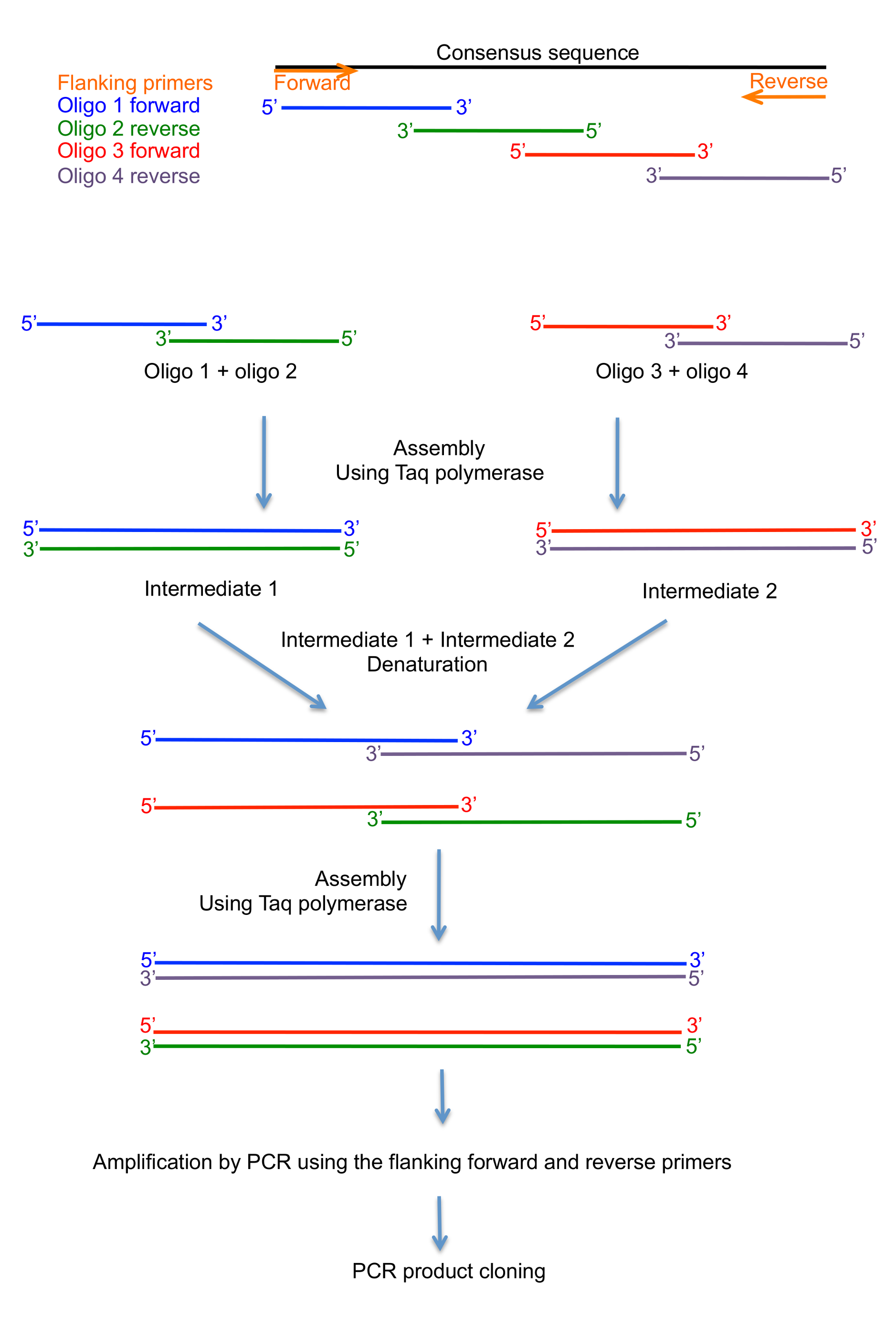

Supplement: Figure S1 — Strategy for the cloning of subgenogroup consensus sequences. Assembly of 4 overlapping oligonucleotides using Taq polymerase then PCR amplification. (TIF) [file pone.0089963.s002.tif]
