# Supplementary material for: SYBR Green Real-Time PCR for the Detection of All Enterovirus-A71 Genogroups
Source: PLoS One. 2014 Mar 20;9(3):e89963. doi: 10.1371/journal.pone.0089963 (PMC3961242; doi:10.1371/journal.pone.0089963)
Supplement: Table S1 — Sequences of the oligonucleotides used for the construction of the twelve subgenogroup specific plasmids. (DOCX) [file pone.0089963.s003.docx]

**Table S1. Sequences of the oligonucleotides used for the construction of the twelve subgenogroup specific plasmids.** The *NotI* restriction site is shown underlined in the third oligonucleotide.

| subgenogroup | oligonucleotide | 5’-3’ sequences |
| --- | --- | --- |
| A | 1 | GAGAGCTCTATAGGAGATAGTGTGAGTAAGGCCCTCACCCAAGCTTTACCTGCACCCACA |
|  | 2 | CCGCTATAATTTTTCCAGTGTCTAAGCGATGACTGCTCACTTGGGTGTTTTGGCCTGTGGGTGCAGGTAAAGC |
|  | 3 | CTTAGACACTGGAAAAATTATAGCGGCCGCTTATTACGAAATCGGAGCTTCGTCGAATGCTAGTGATGAGAGTATGA |
|  | 4 | AGCTGTGCTATGTGAGTTAAGAACACACCGAGTCTCAATCATACTCTCATCACTAGCATTCGAC |
|  | Forward primer | GAGAGCTCTATAGGAGATAGTGTGAG |
|  | Reverse primer | AGCTGTGCTATGTGAGTTAAGAAC |
| B0 | 1 | GAGAGTTCTATAGGGGACAGTGTGAGTAGAGCACTCACCCAGGCCCTGCCAGCACCCAC |
|  | 2 | CGCTATAATTTTGCCAGTGTCCAGTCGGTGACTGCTCACCTGTGTGTTCTGGCCTGTGGGTGCTGGCAGG |
|  | 3 | TGGACACTGGCAAAATTATAGCGGCCGCTTATTATGAAATTGGGGCGTCGTCAAATGCTAGTGACGAGAGCAT |
|  | 4 | TGCTGTGCTATGTGAGTTGAGAACGCATCGCGTTTCAATCATGCTCTCGTCACTAGCATTTG |
|  | Forward primer | GAGAGTTCTATAGGGGACAGTGT |
|  | Reverse primer | TGCTGTGCTATGTGAGTTGAG |
| B1 | 1 | GAGAGTTCTATAGGGGACAGTATGAGTAGAGCACTTACTCAGGCCCTGCCAGCACCCAC |
|  | 2 | CGCTATAATTTCGCCAGTATCCAGTCGATGACTGCTCACCTGTGTGTTTTGACCTGTGGGTGCTGGCAGG |
|  | 3 | CTGGATACTGGCGAAATTATAGCGGCCGCTTATTATGAAATTGGGGCATCGTCAAACACTAGTGACGAGAGTATG |
|  | 4 | TGCTGTGCTATGTGAGTTAAGAACGCATCGTGTTTCAATCATACTCTCGTCACTAGTGTTTG |
|  | Forward primer | GAGAGTTCTATAGGGGACAGTATGA |
|  | Reverse primer | TGCTGTGCTATGTGAGTTAAGAAC |
| B2 | 1 | GAGAGCTCTATAGGAGATAGTGTGAGTAGAGCACTTACCCAGGCCCTGCCAGCACCCAC |
|  | 2 | CGCTATAATTTCGCCAGTATCCAGTCGATGACTGCTCACCTGTGTGTTTTGACCTGTGGGTGCTGGCAGG |
|  | 3 | CTGGATACTGGCGAAATTATAGCGGCCGCTTATTATGAAATTGGGGCATCGTCAAATACTAGTGACGAGAGTATGA |
|  | 4 | TGCTGTGCTGTGTGAGTTAAGAACGCATCGTGTTTCAATCATACTCTCGTCACTAGTATTTGAC |
|  | Forward primer | GAGAGCTCTATAGGAGATAGTGTGAG |
|  | Reverse primer | TGCTGTGCTGTGTGAGTTAA |
| B3 | 1 | GAGAGCTCTATAGGAGATAGTGTGAGTAGAGCACTTACCCAGGCCCTGCCAGCACCCAC |
|  | 2 | CGCTATAATTTCGCCAGTGTCTAGTCGATGACTGCTCACCTGTGTGTTTTGACCTGTGGGTGCTGGCAGG |
|  | 3 | CTAGACACTGGCGAAATTATAGCGGCCGCTTATTATGAAATTGGGGCATCGTCAAATACTAGTGATGAGAGTATGAT |
|  | 4 | TGCTGTGCTGTGTGAGTTAAGAACGCACCGTGTTTCAATCATACTCTCATCACTAGTATTTGACG |
|  | Forward primer | GAGAGCTCTATAGGAGATAGTGTGAG |
|  | Reverse primer | TGCTGTGCTGTGTGAGTTAA |
| B4 | 1 | GAGAGCTCTATAGGAGATAGTGTGAGTAGGGCACTTACCCAGGCCCTGCCAGCTCCAAC |
|  | 2 | CCGCTATAATTTCACCAGTGTCTAGTCGATGACTGCTCACCTGCGTGTTCTGACCTGTTGGAGCTGGCAGGG |
|  | 3 | CTAGACACTGGTGAAATTATAGCGGCCGCTTATTATGAAATTGGGGCATCGTCAAATACTAGTGATGAGAGTATGAT |
|  | 4 | TGCCGTACTGTGTGAATTAAGAACGCATCGTGTCTCAATCATACTCTCATCACTAGTATTTGACG |
|  | Forward primer | GAGAGCTCTATAGGAGATAGTGTGAG |
|  | Reverse primer | TGCCGTACTGTGTGAATTAAGAA |
| B5 | 1 | GAGAGCTCTATAGGAGACAGTGTGAGTAGGGCACTCACCCAGGCCCTGCCAGCACCCAC |
|  | 2 | CGCTATAATTTCACCGGTGTCTAATCGATGGCTGCTCACCTGTGTGTTTTGACCTGTGGGTGCTGGCAGG |
|  | 3 | TAGACACCGGTGAAATTATAGCGGCCGCTTATTATGAGATCGGGGCATCATCAAATACTAGTGATGAGAGTATGATTG |
|  | 4 | TGCTGTACTGTGTGAGTTAAGGACGCATCGTGTCTCAATCATACTCTCATCACTAGTATTTGATGA |
|  | Forward primer | GAGAGCTCTATAGGAGACAGTGT |
|  | Reverse primer | TGCTGTACTGTGTGAGTTAAGG |
| C1 | 1 | GAGAGTTCTATAGGGGATAGTGTGAGCAGAGCTCTCACCCAAGCTTTACCAGCACCCACA |
|  | 2 | CGCTATAATTTTACCAGTGTCCAACCGGTGGCTGCTTACTTGCGTGTTTTGGCCTGTGGGTGCTGGTAAAGC |
|  | 3 | TGGACACTGGTAAAATTATAGCGGCCGCTTATTATGAAATTGGAGCATCATCAAATGCTAGTGACGAGAGTATGA |
|  | 4 | AGCTGTGCTGTGCGAATTAAGAACACACCGTGTCTCAATCATACTCTCGTCACTAGCATTTGA |
|  | Forward primer | GAGAGTTCTATAGGGGATAGTGTGA |
|  | Reverse primer | AGCTGTGCTGTGCGAAT |
| C2 | 1 | GAGAGTTCTATAGGGGACAGTGTGAGCAGAGCCCTCACCCGAGCTCTACCGGCACCTACA |
|  | 2 | CCGCTATAATTTTACCAGTATCCAATCGATGGCTGCTTACCTGCGTGTTTTGGCCTGTAGGTGCCGGTAGAGC |
|  | 3 | ATTGGATACTGGTAAAATTATAGCGGCCGCTTATTATGAAATTGGAGCATCATCAAATGCTAGTGATGAGAGTATGAT |
|  | 4 | AGCTGTGCTATGTGAATTAAGAACACATCGCGTCTCAATCATACTCTCATCACTAGCATTTGATG |
|  | Forward primer | GAGAGTTCTATAGGGGACAGTGT |
|  | Reverse primer | AGCTGTGCTATGTGAATTAAGAACA |
| C3 | 1 | GAGAGTTCTATAGGGGATAGTGTGAGCAGAGCCCTTACCCAAGCTCTACCGGCACCCAC |
|  | 2 | GCCGCTATAATCTTACCAGTATCTAATCGATGACTGCTCACCTGTGTGTTCTGGCCTGTGGGTGCCGGTAGAG |
|  | 3 | ATTAGATACTGGTAAGATTATAGCGGCCGCTTATTATGAAATTGGAGCATCATCGAATGCTAGTGATGAGAGCAT |
|  | 4 | AGCTGTACTGTGTGAATTAAGAACACATCGTGTCTCAATCATGCTCTCATCACTAGCATTCG |
|  | Forward primer | GAGAGTTCTATAGGGGATAGTGT |
|  | Reverse primer | AGCTGTACTGTGTGAATTAAGAACA |
| C4 | 1 | GAAAGTTCCATAGGAGATAGTGTGAGCAGAGCCCTCACTCAAGCTCTACCAGCACCCACA |
|  | 2 | CGCTATAATCTTGCCTGTATCCAGTCGATGACTGCTCACCTGTGTGTTCTGACCTGTGGGTGCTGGTAGAGC |
|  | 3 | CTGGATACAGGCAAGATTATAGCGGCCGCTTATTATGAAATTGGAGCATCATCAAATGCTAGTGATGAGAGCATG |
|  | 4 | AGCTGTGCTGTGCGAGTTAAGAACACAGCGTGTCTCAATCATGCTCTCATCACTAGCATTTG |
|  | Forward primer | GAAAGTTCCATAGGAGATAGTGTGAG |
|  | Reverse primer | AGCTGTGCTGTGCGAG |
| C5 | 1 | GAAAGTTCTATAGGGGACAGCGTGAGCAGAGCCCTCACCCAAGCCCTACCGGCACCTAC |
|  | 2 | CCGCTATAATTTTACCAGTGTCTAGTCGGTGGCTGCTTACCTGCGTGTTCTGACCTGTAGGTGCCGGTAGGG |
|  | 3 | CTAGACACTGGTAAAATTATAGCGGCCGCTTATTATGAGATTGGAGCATCGTCAAATGCTAGTGATGAGAGTATGA |
|  | 4 | AGCCGTGCTGTGCGAATTAAGAACACACCGTGTCTCAATCATACTCTCATCACTAGCATTTGAC |
|  | Forward primer | GAAAGTTCTATAGGGGACAGCG |
|  | Reverse primer | AGCCGTGCTGTGCG |
